# What Overweight Women Want From a Weight Loss App: A Qualitative Study on Arabic Women

**DOI:** 10.2196/mhealth.4409

**Published:** 2015-05-20

**Authors:** Aroub Abdulaziz Alnasser, Abdulrahman Saleh Alkhalifa, Arjuna Sathiaseelan, Debbi Marais

**Affiliations:** ^1^King Saud UniversityFood Science and NutritionRiyadhSaudi Arabia; ^2^University of CambridgeCambridgeUnited Kingdom; ^3^University of AberdeenAberdeenUnited Kingdom

**Keywords:** weight loss, focus groups, smartphone, mobile apps, Arabic, qualitative research

## Abstract

**Background:**

Overweight and obesity are international public health issues. With mobile and app use growing globally, the development of weight loss apps are increasing along with evidence that interventions using technology have been effective in the treatment of obesity. Although studies have been conducted regarding what content health professionals would recommend within weight loss apps, there are limited studies that explore users’ viewpoints. There is specifically a paucity of research that takes the cultural background of the user into consideration, especially in Middle Eastern countries where the lives and weight loss intervention needs of women not only vary vastly from the West, but the obesity rate is also increasing exponentially.

**Objective:**

The current study sought to explore the proposed features of an Arabic weight loss app by seeking the experiences and opinions of overweight and obese Saudi Arabian users in order to design a mobile phone app to fit their needs.

**Methods:**

Focus group discussions were conducted with a purposive sample of volunteer overweight and obese Saudi women (BMI ≥ 25) who were older than 18 years and who owned a mobile phone. The most common Arabic and English weight loss mobile apps were downloaded to initiate dialogue about app usage and to get their opinions on what an ideal weight loss app would look like and the features it would include. All transcribed, translated discussions were thematically analyzed, categorized for each of the main topics of the discussion, and specific quotations were identified.

**Results:**

Four focus groups were conducted with a total of 39 participants. Most participants owned an Android mobile phone and only a few participants were aware of the availability of health-related apps. Barriers to weight loss were identified including: motivation, support (social and professional), boring diets, customs, and lifestyle. Diverse themes emerged as suggestions for an ideal weight loss app including: Arabic language and culturally sensitive; motivational support and social networking; dietary and physical activity tools; and a tailorable, user-friendly interface.

**Conclusions:**

This study identifies weight loss app features from the users’ perspective, which should be considered in the development of a weight loss app for this population.

##  Introduction

Numerous influential studies have shown that overweight and obesity has increased markedly, making it a major international health issue, especially among women. According to the World Health Organization [1], the most recent evidence suggests that women are twice as likely as men to be obese in Europe, the Eastern Mediterranean, and the Americas. This growing trend indicates the need for effective weight control interventions that are accessible to people all over the world regardless of language or location. As the prevalence of obesity around the world has increased, so has the use of mobile technology, particularly in the Middle East and Saudi Arabia [2,3]. This indicates that this technology may be useful as a weight loss intervention. A recent systematic review [4] found that technology interventions, regardless of device type, are an efficient approach for behavioral treatment of obesity in order to achieve or maintain a healthy weight. The recent growth in the development of mobile phone apps has created an opportunity to use them as an intervention tool to both treat and prevent obesity. However, there has been very little research done to discover what users want and need in a weight loss app.

Mobile technology for self-recording and other behavioral modification aspects has been found to be a useful approach to enhancing health [5]. Various apps have been developed for this purpose, but there is evidence that English as well as Arabic apps do not comply with evidence-informed practices for weight management [6,7,8]. Although it is important to ensure that development of an app for weight loss intervention takes these evidence-based weight loss practices into consideration, it is also important to include participants’ opinions and preferences, especially when designing an app for a specific culture. Some research has been conducted to determine the opinion of health professionals relating to weight loss apps; however, there is a paucity of evidence regarding user preferences, which has shown to be crucial in app development for weight loss [9]. This is exemplified by a qualitative study conducted solely with physicians and dieticians in Qatar to develop an obesity management app, which lacked any user contribution [2]. Furthermore, a recent study done on English-language weight loss apps in the UK shows that participants are interested in weight loss apps that provide structure, ease of use, personalized features, and accessibility between devices. However, this study neglected to elect participants who had preexisting overweight and obesity issues, and because the needs of users with existing conditions differ from those who are not suffering from these problems, this cannot be overlooked [10].

This study, therefore, aimed to explore the proposed features for an Arabic weight loss app by seeking the experiences and opinions of overweight and obese female Saudi Arabian users in order to design a mobile phone app to fit their needs.

## Methods

### Participants

A qualitative cross-sectional study was conducted. Focus group discussions were conducted with a purposive sample of volunteer overweight and obese Saudi women (body mass index (BMI) ≥25) who were older than 18 years, owned a mobile phone, and consented to taking part in the study. Women who were pregnant or were diagnosed with chronic diseases of lifestyle—such as cardiovascular disease, hypertension, diabetes mellitus, or cancer—were excluded from the study. Recruitment was done through posters placed around the King Saud University campuses (a public university located in Riyadh, Saudi Arabia, with free tuition that enables students from all segments of society to attend), local shopping malls, on social networks such as Twitter, as well as by word of mouth. The recruitment posters briefly described the study, eligibility criteria, and directed interested participants to complete an online screening questionnaire to ensure compliance with the inclusion and exclusion criteria. Ethical approval was obtained from the ethics committee of the College of Science Research Center of King Saud University. Written informed consent was obtained from all subjects.

### Procedure

A total of 4 focus groups with 6-10 participants in each were planned, with the aim of reaching the point of data saturation until no new information was generated. Those complying with the eligibility criteria were invited to join 1 of 4 possible dates available for focus groups that suited them best. Prior to meeting, an information sheet and consent forms were sent to the participants. Each participant was randomly allocated to one of the most common Arabic/English weight loss apps and asked to download the app before the focus groups.

Focus groups were held in Arabic, within meeting rooms at the women’s King Saud University campus. The focus groups started with a conversation about the participants’ experiences of health-related mobile phone apps in general, following a topic guide that presented open-ended questions to encourage participants to express their opinions. Group management guidelines from Krueger and Casey [11] were followed. The facilitator provided time for the participants to try some of the weight loss apps that they had been asked to download to prompt further discussion. Participants were then asked to describe their thoughts and ideas about the features of these apps. They were asked what they liked, disliked, and would ideally like a weight loss app to have. Their experience of previous weight loss diets was also discussed to identify their opinions of barriers to weight loss.

### Data Analysis

All focus groups were audio-recorded and an observer was available to take notes during the discussions. Refreshments were provided during the focus groups and participants were given a small gift voucher as a thank-you at the end of the focus group. All focus group discussions were transcribed verbatim in Arabic and translated to English. A subsample of the transcripts were then back translated to ensure accuracy. To initiate data analysis, the researchers familiarized themselves with the data by reading all translated transcriptions and identifying themes within each transcript. Thematic analysis was done independently by 2 researchers (AA and DM) for 1 transcript and then jointly for the other 3 transcripts. Once thematic analysis had been completed, specific quotations to support the themes were identified.

## Results

### Participant Characteristics

Four focus groups involving 39 women were conducted. Each group consisted of 7 to 12 participants with a mean age of 29 years (Table 1). Based on self-reported height and weight, the mean BMI was 29.1 with most (67%) of the sample classified as overweight. Marital status was equally represented, and most women had been educated to a tertiary level (77%) and owned an Android mobile phone (56%).

**Table 1:** Demographic characteristics of Saudi women (N=39) involved in the 4 focus groups.

Demographic characteristic		Measurement
Age (years), mean (SD)		29.4 (8)
Height (m), mean (SD)		159.3 (6)
Weight (kg), mean (SD)		73.9 (14)
BMI (kg/m^2^), mean (SD)		29.1 (5)
**Weight classification, n (%)**		
	Overweight (BMI 25-29.9)	26 (67%)
	Obese (BMI 30-34.9)	9 (23%)
	Severely obese (BMI >35)	4 (10%)
**Marital status, n (%)**		
	Single	19 (49%)
	Married	19 (49%)
	Divorced	1 (2%)
**Education level, n (%)**		
	High school	7 (18%)
	Diploma	2 (5%)
	4-year degree	26 (67%)
	Post-graduate	4 (10%)
**Mobile phone brand, n (%)**		
	iPhone	17 (44%)
	Samsung	14 (36%)
	BlackBerry	7 (18%)
	Sony	1 (2%)

### Salient Themes

#### Overview

Themes were categorized for each of the main topics of discussion, namely the experience of apps, barriers to weight loss, and proposals for an ideal weight loss app.

#### Experience of Apps

Few participants were aware of the availability of health-related apps. Only a few participants had previously downloaded a diet app and there was a general lack of awareness of their availability.

I didn’t expect there to be an app concerned about dieting.

Participant 8; Focus Group (FG) 4

Some women in the groups felt that raising awareness of the existence of weight-loss apps in Arabic by providing information or seminars at local clinics and schools would be essential to increasing the knowledge and use of the apps.

When women were asked if they felt they had previously benefited from any health app, similar responses of not knowing about them emerged. If they had downloaded them, it was usually for short periods of use.

I only had the application for 7 days.

Participant 1; FG 3

The most common reason stated for deleting apps was that it did not benefit them. Those that had downloaded weight loss apps indicated that most of them featured meal planners, calorie information, weight loss tips, a water reminder, and most were in Arabic. When women were asked about what health apps they had previously downloaded, they were unable to recall any names.

#### Barriers to Weight Loss

Although a number of women had tried different ways to lose weight, they identified a number of in-common barriers to weight loss including motivation, support (social and professional), boring diets, customs, lifestyle, and misinformation. They also reported poor motivation in initiating the diet, often delaying commencement.

Delaying. I decide to follow a diet on Saturday, and I buy all the diet things that I need on Friday, but I delay it for another 2 days. Then I tell myself that I will start it next Saturday. I have been in that situation for 7 months now.

Participant 6; FG 2

One of the most common issues for women seemed to be a lack of motivation due to inadequate social support.

If I see that no one is with me, I lose interest.

Participant 2; FG 2

They also identified a need for professional health care follow-up as those that had received a dietary prescription often described it as “too boring, restrictive, and frustrating” [Participant 1; FG 1].

Another commonly identified barrier was related to lifestyle, customs, and family obligations such as parties.

We don’t have many entertainment places, so we meet in food places, restaurants, or friends’ houses and everything calls you to eat, as well as girls today are good cooks, they make delicious sweets and you have to eat to keep up with them.

Participant 2; FG 3

From the discussions, it was clear that the majority of participants also had incorrect information or beliefs about weight loss.

You can put 3 bags of salt in your bathtub 3 times a week and that helps lose weight.

Participant 1; FG 3

#### Proposals for an Ideal Weight Loss App

**Figure 1:**
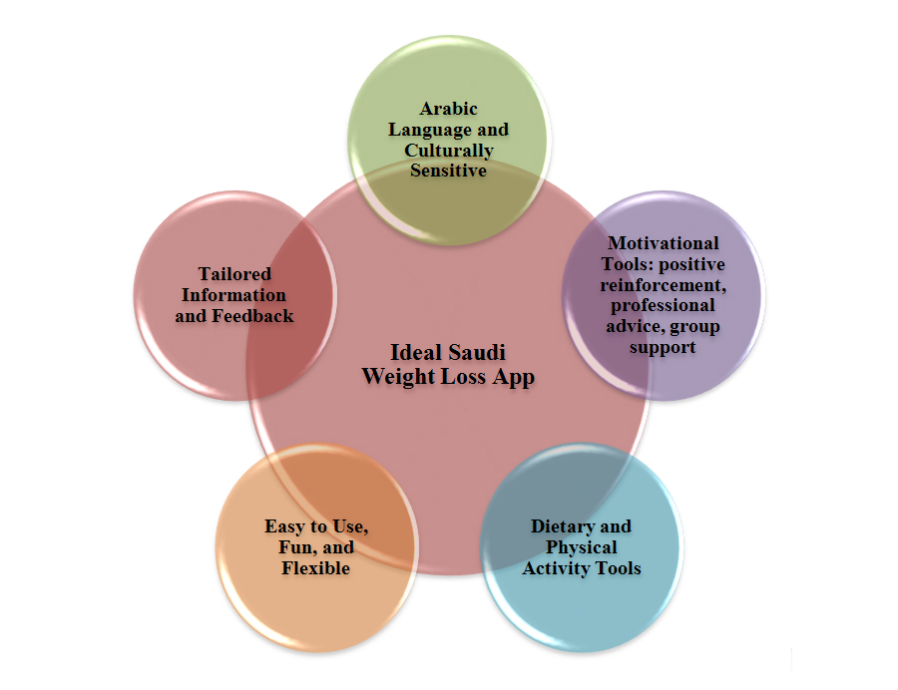
Proposed features for an ideal weight loss app.

Diverse themes emerged from the data as suggestions for an ideal weight loss app for these women. They were classified into 5 categories (Figure 1). The women overwhelmingly indicated that they would prefer an Arabic app.

I have difficulties that relate to the language (when trying the English diet apps).

Participant 3; FG 2

Indeed, it was not only about the ease of language, but also about the language in its social and cultural context. Most women that had previously tried commercial international diet programs felt it would be inconvenient to use them.

I have already subscribed to an American program that … the food was strange for me … we do not eat that because it is not in our culture.

Participant 10; FG 2

I tried to walk 7 days a week but the weather is bad in Riyadh; it is hot, along with shortness of breath and face cover.

Participant 10; FG 2

The most common theme seemed to be the emphasis on motivational support and social networking. The majority of all groups stated that “there has to be communication” [Participant 7; FG 2].

Numerous women indicated that having a social support network would be motivating. For example, one participant said, “The app itself should be like Instagram,” in which women checked up on each other as a group motivator [Participant 1; FG 2].

Having illustrations was also suggested by some participants as a motivator. For example, a participant stated: “An image of a fat woman could appear when the calories consumed exceed the calories expended” [Participant 3; FG 4].

Some of the group members stated that having the ability to ask professionals questions and receive advice regarding their weight loss, in a time sensitive manner, would be greatly beneficial to achieving their weight loss goals.

With regard to dietary and physical activity tools, group members mostly agreed that features such as a BMI calculator, calorie and physical activity counter, weight loss tracker, energy balance calculator, setting goals, vitamin information, and food product information would be useful. Opinions were mixed about planning meals. Some women felt that a meal planner is important and easier than counting calorie intake, but others did not want to follow specific meal plans. However, 2 groups reported that a diet score or competitive games would be encouraging and could be compared if shared with the community.

In terms of layout, participants generally agreed that “it should be simple” [Participant 1; FG 1] and easy to use. They suggested flexible search functions such as “autocomplete” when typing in a search term with results that show the most popular trends. In addition, a barcode reader to lower the burden of adding food items, and an autonomous sensor to monitor physical activity was proposed.

There were various suggestions about controlling the frequency and timing of notifications and tips, such as: “The app (that had been downloaded and used previously) was boring because it reminded me to drink water a lot” [Participant 8; FG 4]. Furthermore, most participants desired tailored notifications taking the users’ previous information and preferences into consideration. Some of the women suggested screening for diseases, age, and dietary preferences (such as being vegetarian) before starting to use the app so that the information and tips provided could be user-specific.

Some expectations such as feedback requirements from the app developer and regular updating of the information were also discussed. Overall, it is clear that the demand for the development of an Arabic weight management app is high, with strong agreement in all 4 groups.

##  Discussion

### Principal Findings

Participants in the study were generally not aware of available health-related apps and had little experience in using them. This is not that surprising since 84% of mobile phone users report having downloaded apps, but only 19% have downloaded a health-related app [12]. Although the use of mobile phone apps in supporting health is promising [13], lack of knowledge of health-related apps might be a reason for the low download rate. Another reason for the low download rate might be the lack of features that Arabic health apps have when compared to English apps. It was found that English-language apps offered on average at least half of the investigated features such as tutorials on use, sharing capability of diet plans, and weight tracking. The Arabic apps only offered 4 of the 12 features mentioned, such as the collection of and information about diet plans and water consumption tracking, substantiating the absence of evidence-based practices. If the available features on the Arabic apps were more attractive to users, then the download rate would likely increase [14].

The Saudi women clearly expressed their preference for the app being in Arabic rather than in English, which is corroborated by the pilot study conducted among health care professionals in Qatar to develop an obesity management mobile phone app [15]. It is also evident that the users of these apps require culturally sensitive information such as locally available foods, physical activities that are possible in their environment, and advice that is specific to the Arabic culture and customs. Parker [16] has indicated that healthy behaviors such as physical activity are boosted or inhibited by custom and culture, so while it may be normal to see Arab men running or exercising at fitness clubs or outdoors, currently this is unusual among Arab women due to cultural restrictions on their activities [17]. A study on morbidly obese Saudi women reported that participants identified factors such as limited social relationships and not being able to practice exercise freely as being the main motivations for choosing surgery over a natural means of weight loss [18].

Most participants attributed negative weight loss experiences to a lack of motivation and social support. As suggested by participants, providing social media features such as Twitter and Instagram would enable participants to share interests, activities, and experiences and thereby provide motivation. Some studies have shown that engagement with Twitter results in greater weight loss [19], while others have found that participants disliked features that permit broadcasting of health-related goals or status updates to friends through social networks [9]. It is suggested that access to social networks be available, but that users have control over what is shared, such as weight loss goals reached or diet scores.

This control over social networking within a weight loss app links to participant suggestions for flexibility and tailoring of the app, where participants felt that they should be able to indicate their preferences for frequency and timing of reminders and notifications they receive, or what features they use. It was clear that participants wanted the app to be easy, user-friendly, and specific to their preferences. The features proposed, such as counters/trackers and goal setting, are consistent with the self-monitoring practices identified in previous studies and would achieve many of the evidence-informed weight loss practices [6-8]. Additional suggestions by participants for food product and vitamin information would address the advice-giving practices.

Many current English and Arabic apps can be described as advice-giving or self-monitoring but lack the behavior modification aspect of weight loss treatment [6-8]. It was interesting that participants proposed features that complied with many of the evidence-informed weight loss practices used by these authors as well as their emphasis on the importance of motivation and social support to evaluate weight loss apps. As many participants had misconceptions regarding weight control, providing evidence-based information or tips is suggested.

### Conclusions

Saudi women were very enthusiastic about the development of a weight loss app that would address their needs. It is clear that cultural sensitivity and social support are seen as the most important aspects to them. This is extremely important as it strengthens the evidence that weight loss interventions need to include some aspect of behavior modification to be effective.

This paper provides unique insights into the views of overweight Saudi women regarding features that might support them in losing weight, giving a voice to the user and informing the development of a weight loss app for Saudi Arabian women. It is suggested, though, that these findings may be relevant to a broader community and should be considered as important aspects for consideration in any weight loss app development process.

## Abbreviations

BMI: body mass index
FG: focus group
